# Exploring breastfeeding support on social media

**DOI:** 10.1186/s13006-018-0166-9

**Published:** 2018-06-15

**Authors:** Nicole Bridges, Gwyneth Howell, Virginia Schmied

**Affiliations:** Western Sydney University, Locked Bag 1797, Penrith, NSW 2750 Australia

**Keywords:** Breastfeeding, Facebook, Online, Social networking sites, Peer support, Ethnography, Authentic presence, Facilitative style, Social network

## Abstract

**Background:**

Lack of breastfeeding support is often cited by mothers as one of the key reasons for premature weaning. The experiences and perceptions of breastfeeding mothers in a range of contexts and their support needs have been studied, but there has been little exploration of the specific breastfeeding topics that women are investigating via social networking sites (SNSs) such as Facebook, and how breastfeeding peer supporters respond to queries about breastfeeding concerns on a SNS.

**Methods:**

This online ethnography took place in the Australian Breastfeeding Association’s (ABA) closed Facebook groups. These groups have been created for breastfeeding mothers to seek and provide support to their peers. All wall posts, comments and images for 15 of these groups were captured over a four-week period between 21 July and 17 August 2013.

**Results:**

The data were collected on a total of 778 wall posts with a total of 2,998 comments posted into the initial wall posts. Analysis revealed that 165 (21%) of these wall posts were queries and 72 (44%) of the queries were specific breastfeeding questions. Twelve breastfeeding topic areas were identified, and the top three topic areas were further analysed for not only their content but the nature of informational and emotional support provided to the community members.

**Conclusions:**

The closed Facebook groups hosted by the ABA provided both informational and emotional support that appeared to be facilitated by an authentic presence from both trained peer breastfeeding counsellors and other mothers. The group administrators played a vital role in both responding to the queries and overseeing the discussions to ensure they adhered to the ABA’s Code of Ethics.

## Background

Breastfeeding is an important public health intervention that impacts significantly on both mothers and their babies [1]. Its importance is reinforced by the World Health Organization’s (WHO) recommendation of exclusive breastfeeding for around 6 m and for breastfeeding to continue for 2 years and beyond [2].

Health professionals have an important role to play in educating and supporting mothers to not only initiate but also maintain breastfeeding for the recommended duration. Consequently, WHO and the United Nations International Children’s Emergency Fund (UNICEF) have implemented the Baby Friendly Hospital Initiative (BFHI) now known in Australia as the Baby Friendly Health Initiative [3]. A recent review of the BFHI found support interventions can increase the duration and exclusivity of breastfeeding, especially face-to-face visits. Similarly, it was identified that education and support delivered by both health professionals or peers can increase breastfeeding initiation rates [4].

Families and friends also play their part [5–11]. In fact, some women indicate a preference for peer support provided by a trained volunteer [12–14]. Lack of breastfeeding support is often cited by mothers as one of the key reasons for premature weaning [13, 15–21] and the need for effective and sensitive professional and social support is key to breastfeeding success [10, 22].

The experiences and perceptions of breastfeeding mothers in a range of contexts have been studied by qualitative researchers [7, 14, 23–25]. However, this paper aims to investigate the specific breastfeeding topics that women are investigating via social networking sites (SNSs) such as Facebook, and how breastfeeding peer supporters respond with informational and emotional support to queries about breastfeeding concerns on a SNS.

Peer support is defined as the “provision of emotional, appraisal, and informational assistance by a created social network member who possesses experiential knowledge of a specific behaviour or stressor or similar characteristics as the target population” [26]. The term ‘created social network’ means the peer supporters are not known to a mother through her informal social network, but rather known to her for the specific purpose of providing support. In the case of this study, peer supporters are known to her because they are members of the same SNS. The online environment of a mother’s group creates a female-dominated community of support. Women use their own experiences of mothering to help themselves and one another [27]. They provide informational and emotional support such as exchanging information, and providing encouragement, empathy and examples of similar experiences [28, 29]. Although there is adequate research exploring the potential of SNSs such as Facebook as an effective tool for health communication [30–34], none of the research specifically analyses its use in relation to breastfeeding.

The study results presented in this paper form part of a larger study investigating how breastfeeding mothers experience online support using closed Facebook groups hosted by the Australian Breastfeeding Association (ABA), a not-for-profit organisation that provides information and support via trained, volunteer peer supporters.

## Methods

In addition to being hosted by the ABA, the SNSs investigated in this study are administered and moderated by trained peer breastfeeding counsellors. Ethnography is a qualitative research method whereby researchers immerse themselves in a community of people to observe their activities, to listen, and to ask relevant questions [35]. The origins of ethnography can be found in anthropology – the methodology used for observing and writing about specific cultural groups [36].

With the proliferation of SNSs including Facebook, communities now refer not only to people in a shared physical location but also to groups of people who congregate online [37]. Consequently, ethnographers are finding that in order to understand how communities work, they must follow these communities onto the Internet and other technologically-mediated avenues of communications. In response to this migration to the digital space, ethnographers have invented some innovative ways to describe these online ethnographies over time. One such term is ‘netnography’, which originated in the area of marketing and consumer research, and incorporates a range of research fields, such as anthropology, sociology, and cultural studies [38]. Netnography is the name given to a “specific set of related data collection, analysis, ethical and representational research practices, where a significant amount of the data collected and participant-observational research conducted originates in and manifests through the data shared freely on the Internet, including mobile applications” [38].

The online ethnography took place in ABA’s closed Facebook groups. These groups have been created for breastfeeding mothers to seek and provide support to their peers. Individual users can ask for support in one wall post and provide support by commenting on another. Therefore, individual users may be both support seekers and providers.

### Recruitment of participants

An online invitation was distributed in early 2013 via ABA email and Facebook inviting groups to nominate for participation in the project. The final 15 groups selected met the criteria as ABA closed groups that were the most active, posting more than once per week. Contact was made with the main administrator of each group who then consulted the group members and distributed participant information sheets about the project. Consent was provided by each group administrator to access the Facebook group and information was posted when this would be occurring so if members did not want to post during that time, they could choose not to do so. At the time of data collection, there were 1846 group members among the 15 groups.

### Data collection

The data collection for this netnography was undertaken by Author 1 following the activities of 15 individual ABA sanctioned closed Facebook groups. All wall posts and comments as well as images for these groups were captured over a four-week period between 21 July and 17 August 2013 and stored electronically in a password-protected file. This data is still relevant, as the ABA continues to utilise Facebook groups to engage with its members and the popularity of their closed Facebook groups has increased substantially since data collection occurred. Also, the ABA’s method of mother-to-mother informational and emotional support remains unchanged.

### Data analysis

The coding scheme was developed drawing on the published study of health networking sites [39, 40]. All wall posts and comments were initially coded according to whether the purpose of the poster was to ask for (‘query’) or share (‘share’) information or emotion. The queries were further grouped into topic areas based on the focus of the support and information the members were seeking and providing. These topic areas are breastfeeding, parenting and the ABA (these posts were about the ABA and its activities). Furthermore, those that were identified as being a query about breastfeeding were then categorised into breastfeeding topic areas.

### Ethics approval

Approval for the study was provided by the ABA and Western Sydney University, Human Research Ethics Committee (approval number H9010). Permission was sought from the administrator of each of the Facebook groups to observe over the four-week period and information was posted and pinned at the top of each of the Facebook groups informing members of the study. Due to the large size of each of the Facebook groups, it was too difficult to orchestrate signed consent forms from each member. If members did not want their posts captured they had the option to not post during that four-week period.

### Reflexivity

Author 1 is currently an active member of the ABA and has held office bearer positions. Authors 2 and 3 have also been members of ABA in the past. This may have influenced the way in which data has been analysed and presented. The research teams were conscious of their position throughout data collection and analysis, and author one prepared reflexive field notes during data analysis which were discussed with the team.

## Results

The data was collected on a total of 778 wall posts with a total of 2998 comments posted into the initial wall posts. This dataset included all wall posts and comments across the 15 groups over the four-week period. Analysis revealed that 165 (21%) of these wall posts were queries and 72 (44%) of the queries were specific breastfeeding questions. Twelve breastfeeding topic areas were identified, and a description of each can be found in Table 1.

**Table 1:** Coding Scheme for Topics of Breastfeeding-related Queries

Code	Category	Description
1	Breastfeeding and exercise	Issues associated with exercising while breastfeeding, including breast refusal
2	Breastfeeding and health	Protective benefits of breastfeeding, breastfeeding during baby’s illness, mother’s diet while breastfeeding, illness while breastfeeding, food intolerance, dieting, menstruation
3	Breastfeeding and medication/drugs/alcohol	Safety of mother taking medication, drugs and alcohol while breastfeeding
4	Breastfeeding and pregnancy	Issues associated with breastfeeding while pregnant
5	Breastfeeding and tongue tie	Tongue tie issues, including poor attachment, low weight gains, reflux, unsettled babies
6	Breastfeeding and work	Breastpump hire, expressing and storing breastmilk, caregivers and the breastfed baby, leaving breastfed babies for prolonged periods
7	Breastfeeding high needs babies	Babies with chronic breastfeeding and behavioural issues such as frequent feeding, poor sleeping, fussiness, intolerance issues, general unhappiness
8	Breastfeeding management	Physical management of breastfeeding, including timing and frequency of feeds, feeding to sleep, breast refusal (inc. distracted breastfeeders, biting and pinching while breastfeeding), breastfeeding with large breasts, positioning and attachment, mastitis, blocked ducts, cysts, white spot, thrush, sore and damaged nipples
9	Breastmilk sharing	Wetnursing, donation of human milk
10	Low supply	Issues associated with low breastmilk supply, including complementary feeding
11	Weaning	Weaning babies from the breast, including reluctant weaners and older children

### Most asked about breastfeeding topics

Of the 72 queries that were specific breastfeeding questions, 55 (76%) were categorised into these three topic areas:

1. Breastfeeding management
2. Breastfeeding and health
3. Breastfeeding and work

These three topic areas are further explained in Table 1 and are discussed below, along with quotations that illustrate participants’ queries and how the online community responded to these queries. The remaining 24% of queries were related to parenting and the ABA and were not analysed for this article.

### Breastfeeding management

The majority (42%) of breastfeeding queries were coded as ‘breastfeeding management’. This topic included areas of discussion such as the physical management of breastfeeding, including timing and frequency of feeds, feeding to sleep, breastfeeding with large breasts, breast refusal, positioning and attachment, mastitis, blocked ducts, cysts, white spot, thrush, and sore and damaged nipples.

Many women were searching for reassurance about the way they were managing their breastfeeding relationship, including breastfeeding to sleep which appeared to be an emotive topic for parents. This mother was seeking information, in addition to emotional support about continuing to breastfeed her baby to sleep.

Boobing to sleep. Quick! Help reassure me it’s normal! Had a moment of craziness this morning and tried to not do it. I continually doubt myself! (P186).

This query yielded 19 responses from other mothers that provided both informational and emotional support. Overwhelmingly, these responses provided encouragement and were ‘normalising’ practices related to breastfeeding by reassuring the mother that breastfeeding to sleep was a normal parenting practice and would not lead to negative outcomes for mother and child.

It’s normal normal normal. I’m typing this as I feed ****** to sleep! It’s easier, it’s comforting, its safe, bonding, beautiful and OKAY!! (P36).

Breast refusal was another frequent topic of discussion within the closed Facebook groups. It is a topic that can be very distressing for breastfeeding mothers, as there is no ‘one size fits all’ approach to solving it, and often the behavior can suddenly disappear without any solution to the puzzle of why it was occurring in the first place.

Below is a query about breast refusal from a mother who is concerned about the issue and reaching out for information, and emotional support in the form of reassurance.

Hi everyone. I’m looking for some advice. My son is 16 months and has been refusing to feed on my left breast for a week or so now. That was all fine until last night when he started fussing when trying to feed off my right breast too. He would latch on for a minute or two and then either bite my nipple or unlatch and cry. I’m very worried my milk supply may be dwindling... He just got him bottom molars on each side so it may be teething related also. Does anyone have any advice for me? Is he weaning? Or am I just not making enough? (P441).

This post generated plenty of comments and a robust discussion about breast refusal and the possible causes. Like this detailed advice below from a peer breastfeeding counsellor in training, most of the responses contained not only realistic and detailed information but some emotional support, including empathy, reassurance, affirmation and encouragement.

Lots of good suggestions there already, ****. Breast refusal can be a really stressful time for you and for bub, but in most cases it is - thankfully - temporary. Lots of no-pressure opportunities to feed can be good - so just hanging out and having lots of skin-to-skin cuddles like ***** mentioned; trying the rocking and singing technique already suggested:); maybe having a nice relaxing bath together; those sorts of things can help bub to feel a bit more like having a feed. Some mums find that offering a feed when their baby is asleep or very drowsy can also mean less chance of refusal, too. If it’s something going on with bub - i.e. teething, or a cold/sore throat, or even an ear infection - then figuring out what it might be and trying to alleviate any discomfort (e.g. giving bub something cold to chew on before a feed; seeing a GP if you suspect something else might be going on, and starting treatment if needed) often helps get breastfeeding back on track, as well. And if it is your menstrual cycle or ovulation affecting the milk in some way, rest assured that’s temporary as well! :) (P465).

The information and support provided by the peer breastfeeding counsellor in training and others as a response to this mother’s post appeared to achieve the goal of providing reassurance and comfort to the mother, as indicated by her final reply.

Such a huge sigh of relief on my end!! I’ve heard that feeding off one breast can still work. Thanks for clarifying for me! Thanks ladies you are awesome:) (P441).

### Breastfeeding and health

Another popular topic was ‘breastfeeding and health’ (19% of breastfeeding queries). This included breastfeeding queries about the protective benefits of breastfeeding, breastfeeding during baby’s illness, mother’s diet while breastfeeding, illness while breastfeeding, food intolerance, dieting, and menstruation.

The following is a condensed example of a considerably long query about breastfeeding and health from a mother who indicated she was experiencing a high level of guilt about her breastfed baby’s health. This is an example of when strong emotions are embedded in a post, and a participant seeking emotional support is reaching out for reassurance. Her introduction provides a brief informational description of how the illness began, including the severity of the illness.

My husband brought a nasty cold virus to our house last week and **** started having the symptoms of cold about Wednesday and we went to GP (on Friday) when her mucus turned greenish as we thought it was an infection… (P383).

The mother goes on to describe her emotional distress about her baby’s illness, and the guilt and anxiety she is feeling about having to give her baby antibiotics after fighting so hard to breastfeed.

It’s ****’s first cold/bacteria/antibiotic in her 9.5 months and it breaks my heart so bad that she has it that early in her life. I know antibiotics kill some good bacteria as well as bad ones, I fought a lot in the last 9.5 months with low supply and how to increase it….. (P383).

She appears worried that the antibiotics are going to compromise the baby’s immune system, and is feeling anger toward her partner for bringing the illness home. She feels like she has failed her baby and needs reassurance that her baby will be OK.

What breaks my heart is apart from all the hundreds of benefits she gets from my milk, my biggest aim was my baby to build a great immune system and I feel like it’s already wrecked :(I played the blame game for 2 days and didn’t talk to my husband because he was sick and passed it to **** and now I feel like crying because she is on antibiotics and I failed on building a good immune system for my baby. (P383).

The online community displayed an overwhelming response to this mother’s cry for help. There were many responses, and the majority of them were focused on providing emotional support, empathy, affirmation, and reassurance – far more than simply providing answers to questions. Interestingly, the following response is from a trained peer breastfeeding counsellor. Her response demonstrates positive appraisal and a justification for the mother having to administer antibiotics to her baby. She also reframes the situation by highlighting how much of an advantage she has already provided for her baby by breastfeeding thus far, and goes on to provide a personal example. Her response also contains informational support in the form of realistic, accurate and sufficiently detailed information that encourages breastfeeding.

I’d say a big pat on the back for building a lovely immune system in your daughter thus far – if she had been bottle fed, she probably would have had more infections prior to this one. This other thing is that breastfed babies recover so quickly and are usually a lot less sick than their formula-fed counterparts. My first child had antibiotics twice in her first year (once intravenously) my second child didn’t have any for 5.5 years and my third hasn’t had any yet and he’s 3. At age nearly 12 my daughter’s immune system is as good as her brothers’ and is rarely away from school. I credit it to the 1.5 years of breastfeeding her! (P96).

The following is an example of another response to this mother, but from a member who is not a trained peer breastfeeding counsellor.

I have a couple if things to add to this too. Firstly you are being super hard on yourself. Remember that we are all human and are susceptible to getting sick that’s part of being alive. It’s winter and cold. A Doctor friend told me that statistically kids will have about 15 sicknesses in the first 5 years. Imagine a world without antibiotics. Lastly your daughter is so so so lucky to have a mum who takes her responsibility as a mother so thoughtfully and seriously. I hope all your peeps recover quick sticks. (P19).

It is evident that the online community was meeting the emotional needs of this mother as she provided feedback in the form of several responses to the Facebook comments.

You’re so lovely ****** that you actually made me smile:) thank you:) (P383).

The following is a post from a mother who has been sick herself. On the surface, she seems to be only seeking informational support about the impact of the illness on the quality and quantity of the breastmilk she is producing for her four-month old baby. She is clearly quite concerned as she asks if she should return to hospital.

I’ve been really sick for the past week - I think I caught gastro from one of the step kids... Anyway, I haven’t been able to keep down any solids until today but it doesn’t seem to have impacted my milk supply as my son (4mo) has still been feeding really well - practically 24/7. How frequently should I be eating and what are some good meals I should start having to build up the goodness in the breastmilk again because all I’ve had these past 5–6 days is water. Should I go back to hospital days and be eating the three course meals for breaky, lunch and dinner? (P522).

Although this seems to be an informational query, the following comment from a trained peer breastfeeding counsellor indicates that the respondent has sensed the mother may also need some emotional support, because she has been quite ill and is possibly concerned about the impact of her illness on her baby. This response starts by reassuring the mother about the quality and quantity of her breastmilk, then provides some practical and detailed suggestions for looking after herself and points the mother to specific resources to meet her informational needs. She finishes with an offer of practical help if the mother needs it and a good wish in the form of kisses.

*****, your breastmilk still has all the goodness in it - if your supply hasn’t been impacted at all then that’s great. Just try to look after yourself, slowly getting some good meals into you. There’s an article on the ABA website about breastfeeding when you’ve got gastro and I cannot for the life of me find it at the moment. Gah! Can anyone else see it???? Make sure you keep hydrated too. It’s often the case that when we are unwell, our supply and breastmilk is absolutely fine but unfortunately our bodies take the hit - I guess it’s a way of protecting our babies from whatever it is we have got!!:) Hugs lady, let us know if we can do anything for you xx (P330).

### Breastfeeding and work

Many women in these online communities were seeking advice and support about ‘breastfeeding and work’. This included discussion about breast pump hire, expressing and storing breastmilk, caregivers and the breastfed baby, and leaving breastfed babies for prolonged periods of time. The majority of queries under this topic were informational, but some mothers still demonstrated a need for emotional support. The following post demonstrates a mother seeking practical information about what food and drink to prepare for her breastfed baby when she returns to work, while at the same time seeking emotional support as she has admitted to feeling nervous about leaving her baby, who appears to be unexpectedly reluctant about drinking expressed breastmilk.

Very nervous about working next week. It is for a few hrs in ** ********. I can either leave **** with hubby and EBM and hope for the best, leave her with hubby and formula and hope for the best, leave them with both + solids and hope something works, or have the whole family come to ** ******** with me so I can feed her before and after work. I really didn’t imagine when I accepted this work months ago that she might not take EBM. I thought by 6.5 months she’d be set. Any ideas? (P170).

This query received only one response from a group member who was also a trained peer breastfeeding counsellor. She provides the mother with reassurance and reminds her that her baby is being cared for in a safe and loving environment. She follows up the emotional support with some realistic and sufficiently detailed information about how to ensure the baby is well hydrated and other ways for her husband to distract the baby in her absence. It was such a thorough and comprehensive answer that it seemed no other members of the online community felt the need to add to the response.

She’ll be fine, enjoy your day! Hubby will cope with whatever decision you make and if she doesn’t take much, nothing bad will happen, she is safe and cared for! Maybe verse him on only warming up very small amounts and he could always cup feed it so she stays hydrated. And if she’s liking solids, that could always be a backup to distract her. (P96).

Often the queries were ‘last minute’ as many mothers returned to work unexpectedly or left their breastfeeding preparation to the last minute. Queries like this one demonstrate that the mother is in need of some emotional support as she has been caught off-guard and is also seeking reassurance that her baby will be alright.

I’m going back to work tomorrow after 3 years. Ten month old bub will have to go cold turkey. Hubby will be home with her. Have cooked up her pumpkin and expressed a bit. Only found out I was going to work earlier today so didn’t get a chance express more. She will be ok right? (P120).

There were many messages of support in reaction to this mother’s post. The majority of them simply wished her well and gently reassured her that her baby would be OK.

Good luck *****! I’m sure she’ll be fine. (P283).

This mother appreciated the support as she provided responses to the comments and provided an update after the event.

She gulped all the milk early morning. I had only 120 mLs ready for her. I am expressing now for her feed tomorrow. She was very pleased to see me this arvo [afternoon].

It’s only a casual job till Friday until something else pops up so that will give me more to to get my head around it and prepare more.

did miss kids heaps though. (P120).

These findings demonstrate the informational and emotional support provided by these online communities.

## Discussion

The first area of study this paper aimed to explore was the specific breastfeeding topics that women are investigating via SNSs such as Facebook. Although there are studies that have explored the most common breastfeeding problems that lead to early cessation of breastfeeding [41–47], there are no studies that specifically explore the most common breastfeeding topics discussed on SNSs.

This study observed 15 closed Facebook groups created for breastfeeding support over a four-week period, explored the 72 posts that were specific breastfeeding queries, and found that 55 (76%) were categorised into the three topics areas of breastfeeding management, breastfeeding and health, and breastfeeding and work. Research conducted in the US into the reasons women stop breastfeeding during the first year, also reports these three topic areas as important in the mothers’ decision to stop breastfeeding [46]. Similarly, a research study conducted in Hong Kong also found that reasons related to breastfeeding management, maternal health and returning to work were cited by mothers as the primary reason for weaning [47]. Mothers in Belgium also reported on the major reasons for early weaning, and breastfeeding management, mother’s and baby’s health, and returning to work were also included in the top nine reasons they stopped breastfeeding [44]. A study carried out in Western Australia discovered that breastfeeding management issues and infant and maternal health issues were common reasons for terminating breastfeeding in the early weeks postpartum (2–10 weeks of age) and returning to work was a common reason when the infant was a little older (19–21 weeks of age) [41]. It is clear that these themes are common in high-income countries and are important areas in which breastfeeding mothers need support to meet their breastfeeding goals.

The second area of study this paper aimed to explore was how breastfeeding peer supporters respond with informational and emotional support to queries about breastfeeding concerns on a SNS, and how these relationships are developed online. Although both trained peer breastfeeding counsellors and other mothers were members of these online communities, it was usually evident which responses were from the trained peer breastfeeding counsellors. Many of the examples presented in the findings were responses from trained peer breastfeeding counsellors and the examples demonstrate why breastfeeding peer support is a key intervention to assist in the improvement of breastfeeding rates [10, 24].

In a thematic analysis of the same cohort, members of three of the closed Facebook groups participated in online depth interviews and online semi-structured focus groups [40]. The overarching theme identified was support, with four sub-themes that describe the nature of online breastfeeding support within the Facebook environment. These sub-themes were: community, complimentary, immediate and information. It was found that the SNSs provided support from the trusted community. It was immediate, it complimented existing support or services that the ABA provide, and also provided practical and valuable information for its users [40].

In a metasynthesis of women’s perceptions and experiences of breastfeeding support, Schmied et al. [48] identified key ideas and concepts that were similar across 31 studies related to breastfeeding support. They identified four major types of breastfeeding support. The most effective was deemed to be ‘authentic presence’ which describes care that women felt was supportive and indicates a trusting relationship and connection or rapport between the woman and peer supporter. There are seven themes within the authentic presence category: being there for me, empathetic approach, taking time touching base, providing affirmation, being responsive, sharing the experience, and having a relationship. In each of the examples of responses presented in the findings, there is evidence of these seven themes in the data.

The second positive type of breastfeeding support identified by Schmied et al. was labelled the ‘facilitative style’ and was strongly associated with an authentic presence. It is defined as ‘an approach to health promotion, or helping, that enables people to draw on a range of information and experience and learn for themselves’ [48]. The facilitative style can be described as a learner-centred approach and comprises five themes: realistic information, accurate and sufficiently detailed information, encouragement for breastfeeding, encouraging dialogue, offering practical help and being proactive. Many of the examples presented in the findings demonstrate this facilitative approach, with the presentation of practical suggestions and informational support alongside the emotional support that is characteristic of the authentic presence approach. This facilitative style was most often seen demonstrated by trained ABA peer breastfeeding counsellors.

### Limitations

This study was conducted on SNSs where the majority of members are committed to and invested in breastfeeding. As the sites were hosted and moderated by trained volunteers of the ABA who are required to abide by a Code of Ethics, negative interactions rarely occurred, compared to other SNSs aimed at mothering and breastfeeding. Also, although it was found that the women in the study found the online environment supportive, the research does not provide any information about whether being a member and engaging in the SNS extended or supported members to continue breastfeeding.

### Future research

Future research could include a comparison of face-to-face breastfeeding support with online support via SNSs and an analysis of the aspects of support we may be losing in the process of moving support online. This could include a detailed exploration of what that means to peer support relationships and the elements included in face-to-face support that may not be possible when supporting women via SNSs.

## Conclusions

This study analysed 15 closed Facebook groups established by the ABA to explore the specific breastfeeding topics that women are investigating via SNSs such as Facebook. Of the 72 queries that were specific breastfeeding questions, 55 (76%) were categorised into these three topic areas of breastfeeding management, breastfeeding and health, and breastfeeding and work which are all themes universally recognised as some of the most common reasons for the cessation of breastfeeding, and important areas for future study.

Secondly, the study explored how breastfeeding peer supporters respond with emotional support to queries about breastfeeding concerns on a SNS. The research found that these types of SNSs facilitated by trained peer breastfeeding counsellors are more likely to facilitate an authentic presence and facilitative style, both recognised as essential components of breastfeeding support.

## Abbreviations

ABA: Australian Breastfeeding Association
BFHI: Baby Friendly Health Initiative
NHMRC: National Health and Medical Research Council
SNS: Social networking site
UNICEF: United Nations International Children’s Emergency Fund
WHO: World Health Organization
